# Low Risk for Marginal Ulcers in Duodenal Switch and Gastric Bypass in a Well-Defined Cohort of 472 Patients

**DOI:** 10.1007/s11695-020-04822-8

**Published:** 2020-07-08

**Authors:** Zakaria Bekhali, Magnus Sundbom

**Affiliations:** 1 Department of Surgical Sciences, Uppsala University, SE-751 85 Uppsala, Sweden; 2 Department of Surgery, Gävle Hospital, Kirurgmottagningen, Gävle sjukhus, SE-801 88 Gävle, Sweden

**Keywords:** Bariatric surgery, Complications, Duodenal switch, Gastric bypass, Marginal ulcer, Incidence rate, Long term

## Abstract

**Purpose:**

Marginal ulcer (MU) is well-known complication in bariatric surgery. Several studies are available in Roux-en-Y gastric bypass (RYGBP), while data on the incidence in duodenal switch (DS) is limited. We aimed to compare the incidence of MU between DS and RYGBP in a well-defined cohort and to identify associative factors.

**Methods:**

A cohort of 732 patients with BMI ≥ 48 who had undergone primary DS or RYGBP during 2008–2018 received a questionnaire concerning ulcers, PPI therapy, and smoking habits; hereafter, patient charts were reviewed. Incidence rates (IRs) for MU were calculated in our survey and on previous registered data in the national quality register for bariatric surgery (SOReg). A multivariate regression analysis was performed to identify predictive risk factors for MU.

**Results:**

After a mean follow-up of 6.1 years, 472 (64%) patients responded (47 ± 11 years old, 65% women and 42% DS). Of 41 MUs identified, 23 were endoscopically verified. Gastrointestinal bleeding, abdominal pain, and dysphagia were the most common symptoms. IR for MU was 1.4% (DS 1.3% and RYGBP 1.5%) per patient-year, compared with 0.9% according to SOReg-data. Persisting PPI treatment was seen in about three quarter of former MU patients (OR 11.2 [3.6–34.7], *p* < 0.001), but no other associative factors were found.

**Conclusion:**

The overall risk for MU was low, about 1% per patient-year, without difference between DS and RYGBP. Ongoing PPI treatment was frequent in many former MU patients. This study on MU after DS provides reassuring results for future bariatric surgery candidates.

**Electronic supplementary material:**

The online version of this article (10.1007/s11695-020-04822-8) contains supplementary material, which is available to authorized users.

## Introduction

Severe obesity is associated with increased mortality and morbidity [1, 2]. Surgical treatment results in sustainable weight loss, reduced comorbidity, and improvement of quality of life [3, 4]. Among several bariatric procedures, gastric bypass (RYGBP) is well-established worldwide [5] and often considered as gold standard. Duodenal switch (DS) usually performed on patients with higher BMI (BMI > 50) results in more sustainable weight loss [6, 7] but is less frequently performed due to increased technical demands and risk for metabolic complications in the long term [8–10]*.*

Despite the benefits in bariatric and metabolic surgery, operated patients will risk some typical complications such as the development of a marginal ulcer (MU) at, or just distal to, the gastroenteral anastomosis. The pathophysiology is multifactorial, and factors like high acidity, for example, due to large pouch size or gastro-gastric fistula, *Helicobacter pylori*; local ischemia; and remaining foreign bodies from staple material as well as the used staple technique in itself have been mentioned in many studies [11–18]. Moreover, several patient-related factors such as smoking, use of corticosteroids and NSAIDs, and various obesity-related comorbidities have also been described [12, 15]. Abdominal pain is most common symptom of MU, whereas other symptoms include gastrointestinal bleeding or anemia and dysphagia due to stricture [15, 17, 19]. The diagnosis of MU is confirmed with upper endoscopy, but a significant proportion of patients with symptoms suggestive of MU are empirically treated by proton pump inhibitors (PPI) [15, 17, 20–22].

The incidence of MU ranges between 0.35 and 16% in RYGBP [11, 23, 24] and seems to be low (0.3%) in DS [7], although only a limited number of studies exist. In a previous study with wireless pH-metry, we were able to show that the duodenal bulb has a buffering effect, reducing the time with pH < 4 from 59% in the gastric sleeve to 14% distal to the pylorus. This could theoretically result in lower incidence of MU at the duodeno-ileostomy after DS [25].

In Sweden, bariatric surgery is registered in a national quality register, the Scandinavian Obesity Surgery Registry (SOReg), and since 2007, SOReg contains more than 80,000 patients. Most of them have had a RYGBP (79.2%), while about 1% have been operated with DS. Data is collected at baseline, perioperatively, and at follow-up at 6 weeks and 1, 2, 5, and 10 years. While coverage has been proven high (97%) [26], long-term follow-up is not optimal, around 50% at 5 years.

This retrospective observational cohort study aimed to determine and compare the incidence of MU in DS and RYGBP patients and to find associative factors for development of MU. Furthermore, we have compared our results with registered SOReg-data.

## Material and Methods

Patients having had bariatric surgery at our centers are entered into a local database as well as in SOReg. Postoperatively, patients are followed at our out-patient clinic at 6 weeks and then annually. Data from the perioperative period and the 1- and 2-year follow-up are entered in SOReg. In total, 778 patients with BMI ≥ 48 having primary DS or RYGBP between January 2008 and September 2018 were identified in local databases. Patients later having had a revisional procedure (*n* = 27) were excluded as were deceased patients (*n* = 15) and those without a known address (*n* = 4) at the time of survey. The 732 selected patients received a paper questionnaire which comprised six questions concerning symptoms, diagnosis, and treatment of postoperative ulcers as well as ongoing treatment with PPI and smoking habits Supplementary Table 1*.* In responding patients, we thoroughly reviewed the patient charts, and when we were not clear about any response, we telephoned the patient to ensure clarity. At baseline, comorbid conditions were defined as ongoing treatment of diabetes, hypertension, dyslipidemia, and sleep apnea. Operative time, surgical access, and postoperative complications within 30 days were noted as well as all clinical information on MU during follow-up. Because of the varying duration from surgery, the incidence rate (IR) of MU was determined after calculating years at risk. SOReg-data, containing registrations from routine clinical care in responding patients, was examined similarly.

### Statistical Analysis

Data for age, BMI, and weight are shown as mean ± standard deviation, unless otherwise noted. Categorical data were analyzed by *χ*2 test, while two-tailed Student’s *t* test was used for quantitative data. *p* < 0.05 was regarded as statistically significant. For both data sets, the years at risk were calculated subtracting the date of surgery from the time of survey or last clinical follow-up in SOReg, respectively. IR of MU per patient per year was calculated by dividing the total number of MU by number of years at risk. To identify predictors for development of MU, we performed univariate and multivariate analysis including clinically relevant baseline and perioperative data and BMI reduction at 2 years, as well as smoking habits and ongoing PPI treatment. To test equality of two incidence rates for MU between survey and SOReg, we used exact tests due to the small numbers of cases and report mid *p* values due to the discreteness of the *p* value. SPSS (IBM, Armonk, NY, USA) was used for all analysis, except the analysis between the two IR which was performed in using STATA (Stata/SE version 15.1 for Windows).

## Results

### Included Patients

In total, 472 patients (64%) answered the questionnaire, whereof 65% were women and 42% had a DS. As demonstrated in Table 1, DS patients had somewhat higher preoperative BMI (56 vs 51) and longer operative time (149 vs 93 min).

**Table 1:** Demographics of the included patients and number of marginal ulcers

	Total	DS	RYGBP	*p* value
Included patients	472	199 (42%)	273 (58%)	–
Women, *n* (%)	308 (65%)	120 (60%)	188 (69%)	0.054
Age at operation (years)	41.1 ± 10.7	40.9 ± 10.3	41.3 ± 10.9	0.640
Preoperative weight (kg)	156.8 ± 23.9	169.6 ± 25.2	147.5 ± 17.7	< 0.001
BMI (kg/m^2^)	53.7 ± 5.4	56.8 ± 6.3	51.4 ± 3.2	< 0.001
Any comorbidity, *n* (%)	309 (65%)	140 (70%)	169 (61%)	0.057
Operative time (min)	117 ± 42	149 ± 29	93 ± 35	< 0.001
Surgical access				< 0.001
Open	248 (52%)	130 (85%)	77 (28%)	
Laparoscopy	215 (45%)	30 (15%)	185 (68%)	
Converted	11 (2%)	–	11 (4%)	
Postoperative complication (30 days)	62 (13%)	28 (15%)	34 (13%)	0.545
At time of survey				
Time from surgery (years)	6.1 ± 2,7	6.1 ± 2.9	6.2 ± 2,5	0.768
Age (years)	47.3 ± 11.1	47 ± 10.9	47.6 ± 11.3	0.602
Smoking, *n* (%)	50 (11%)	21 (11%)	29 (11%)	0.986
Ongoing PPI, *n* (%)	123 (26%)	48 (24%)	75 (27%)	0.413
Marginal ulcers in survey, *n* (%)	41 (9%)	16 (8%)	25 (9%)	
Incidence rate (MU/patient-year)	1.4%	1.3%	1.5%	0.721
Marginal ulcers in SOReg, *n* (%)	17 (2%)	8 (3%)	9 (2%)	
Incidence rate (MU/patient-year)	0.9%	1.1%	0.7%	0.458
Comparison of IR in survey vs SOReg	*p* = 0.084	*p* = 0.643	*p* = 0.067	

Data presented as mean (SD) if not otherwise stated

### Survey

The cohort had a mean follow-up of 6.1 ± 2.7 years. In total, 41 (8.6%) of the patients reported in their questionnaire to have had an MU during the study period, thus resulting in an incidence rate of 1.4% (DS 1.3% and RYGBP 1.5%), without significant difference between the two procedures (*p* = 0.721). As demonstrated in Table 2, gastrointestinal bleeding, abdominal pain, and dysphagia were the most common symptoms leading to endoscopy and the subsequent diagnosis of MU. All 23 ulcers were found on the small bowel side of the anastomosis, and in 4 RYPGBs, a stenosis in the gastrojejunostomy was also diagnosed.

**Table 2:** Symptoms leading to endoscopy and diagnosis of marginal ulcer, as well as any additional diagnoses, in the 23 patients having had gastroscopy

Patient	Abdominal pain	GI bleeding/anemia	Dysphagia	Additional diagnoses	Procedure
1	Yes				DS
2	Yes				DS
3		Bleeding			DS
4		Bleeding			DS
5	Yes	Anemia	Yes	Gastritis	DS
6		Anemia			DS
7		Bleeding			DS
8		Bleeding			DS
9	Yes		Yes		RYGBP
10	Yes				RYGBP
11		Bleeding			RYGBP
12			Yes		RYGBP
13			Yes	GJ stenosis	RYGBP
14	Yes		Yes	GJ stenosis	RYGBP
15	Yes				RYGBP
16		Anemia			RYGBP
17		Bleeding			RYGBP
18			Yes	GJ stenosis	RYGBP
19	Yes	Bleeding			RYGBP
20		Bleeding			RYGBP
21	Yes				RYGBP
22		Bleeding	Yes	GJ stenosis	RYGBP
23	Yes	Bleeding			RYGBP

Median time from operation to diagnosis of MU was 1.9 (0.1–7.6) years in these 23 patients. The remaining 18 patients had got their diagnosis based on clinical symptoms suggestive of MU. All 41 patients had been treated by PPI. At survey, there was no significant difference between the two procedures regarding ongoing PPI treatment (24% vs 27%, *p* = 0.413) nor smoking habits (11% for both). The main indications for ongoing PPI treatment were abdominal pain (50%) and gastroesophageal reflux (24%).

In both the univariate and multivariate analysis, there was a significant association between a former MU and ongoing PPI treatment (OR 11.2 [3.6–34.7], *p* < 0.001). No other associative factors were found Table 3.

**Table 3:** Univariate and multivariate analyses of clinical important factors associative to developing a marginal ulcer

	Univariate analysis			Multivariate analysis*		
	Odds ratio	95% CI	P value	Odds ratio	95% CI	*p* value
Gender	1.31	0.65–2.65	0.442	0.63	0.20–1.97	0.433
Age at surgery	0.99	0.96–1.02	0.602	0.96	0.91–1.01	0.185
BMI	0.97	0.91–1.03	0.405	0.89	0.75–1.07	0.241
Any comorbidity	0.64	0.33–1.23	0.189	0.55	0.16–1.87	0.346
Postop complication	0.76	0.32–2.29	0.767	1.52	0.34–6.76	0.578
Surgical method	0.86	0.45–1.67	0.671	2.48	0.70–8.77	0.158
BMI loss at 2 years	1.02	0.94–1.10	0.562	1.03	0.93–1.14	0.566
Smoking	1.79	0.44–3.15	0.743	0.39	0.04–3.47	0.401
Ongoing PPI	9.70	4.68–20.10	< 0.001	11.27	3.65–34.78	<0.001

*Nagelkerke *R*-square 0.248; 203 cases included

### SOReg-Data

For SOReg-data, the incidence rate for MU in our DS and RYGBP was 1.1 and 0.7%, respectively, with no significant differences between the two procedures (*p* = 0.458). Compared with the results from the present survey, there was a trend towards a lower overall risk for MU (0.9% vs 1.4%, *p* = 0.084) as well as in RYGBP (0.7% vs 1.5%, *p* = 0.067).

## Discussion

In the present cohort of 472 patients with an initial BMI > 48, we were able to show a low incidence rate for marginal ulcers in DS and RYGBP, 1.3% and 1.5%, respectively, during a mean follow-up of 6.1 years. As expected, abdominal pain, gastrointestinal bleeding, and dysphagia were the most common symptoms leading to endoscopy and a diagnosis of an MU. Of the endoscopically verified MU patients, 57% were diagnosed within the first 2 postoperative years. Persisting PPI treatment was seen in 73% of former MU patients, but no other associate factor could be determined.

Our survey is one of the few that describes the incidence of MU after DS. We chose to include only patients with BMI ≥ 48 kg/m^2^, having had primary bariatric surgery without any revisional procedure, to make the two groups as comparable as possible. Because of our clinical patient selection mechanisms, the studied DS patients had somewhat higher BMI, while the longer operative time was due to the use of open surgery in DS at that time. Furthermore, the overall risk of early postoperative complications in the present cohort of patients with elevated BMI is almost doubled (14% vs 7%) compared with all patients having bariatric surgery in Sweden (mean BMI 41) [27].

### Incidence Rate of Marginal Ulcer

The present incidence rate of MU was just above 1%, i.e., only one out of 100 operated patients will suffer an MU annually. As mentioned earlier, Hess et al. described an incidence of 0.3% under a follow-up period of over 10 years in DS, whereas Pata and Scopinaro demonstrated a higher incidence of MU in classic biliopancreatic diversion, 2% and 3.2%, respectively [28, 29]. The difference could perhaps depend on the larger gastric remnant in biliopancreatic diversion, resulting in a higher amount of gastric acid passing through the upper anastomosis. This theory is supported by an earlier study by our group on RYGBP, in which we were able to show that the time with pH < 4 in the gastrojejunostomy was longer in patients with MU (69%) compared with in non-MUs (20%, *p* < 0.01) [14]*.* Interestingly, these absolute numbers are very similar to the time with pH < 4 in non-symptomatic DS patients, 59% in the acid-producing sleeve and 14% in the buffered duodenal bulb, as mentioned in the introduction. It would therefore be of great interest to measure the acidity in the present patients with MU. In the background population, the lifetime prevalence of a peptic ulcer is estimated to be 5–10% with an incidence of 0.1–0.3% per year [30, 31]. Thus, the risk for having an ulcer is ten-fold in patients with a history of bariatric surgery. The majority of MU presented within the first 2 years, which is in line with several other studies [17, 19, 32]. Furthermore, none of our patients suffered a repeated MU nor a perforated MU, which otherwise is reported in 0.25 to 1% of cases [33].

### Difference Between our Survey and SOReg-Data

Compared with SOReg-data, the present survey had a trend towards higher overall incidence rate for MU (1.4% vs 0.9%, *p* = 0.084) as well as MU in RYGBPs (1.5% vs 0.7%, *p* = 0.067). This is probably due to more detailed data obtained by our questionnaire and the fact that SOReg-registrations are made in routine clinical care. Moreover, the diminishing follow-up rate of our patients in SOReg (38% and 21% at 2 and 5 years, respectively) reduces the possibility of having a registered MU.

### Ongoing Treatment with PPI

PPI treatment leads to complete resolution in all MUs. Persisting PPI treatment was seen in almost three quarters of our former MU patients, thus highly associated to MU (OR 11.2 [3.6–34.7], *p* < 0.001). This high number indicates either an increased vulnerability to acid-related conditions or a concern among patients and physicians of having a repeated ulcer. Furthermore, a quarter of the patients in the present cohort had ongoing PPI treatment, which is higher than in an earlier national study by our group on gastrointestinal symptoms 5 years after bariatric surgery. In that study, we were able to verify the good anti-acidic effect in RYGBP as the use of PPI was significantly reduced postoperatively, from 9.9 to 6.4% of the patients [34]. However, in general practice (*n* = 40,000 patients), it was found that a surprisingly large proportion, 14.7%, were PPI users and the author concluded that a substantial number of individuals did not meet the correct indications for prescription [35]. Finally, in minimizing the risk for MU development in bariatric surgery, there is an increasing trend to administer PPI as a postoperative prophylaxis. However, neither a clear consensus on the duration of such treatment (30 or 90 days) nor solid evidence of its effect in the long term exists [20, 22, 36, 37].

### Limitations

The well-defined cohort and thorough review of patients’ charts is a strength of the present study, thus adding to the limited number of studies on MU in DS. Although the number of patients (*n* = 472) is a limitation, the present work provides a more accurate incidence rate of MU than in former registry studies. The response rate was not optimal (64%); however, in a sensitivity analysis, responders only differed from the remaining patients by being older (41 vs 34 years) and more often having comorbidities (65% vs 55%, *p* < 0.05 for both). Moreover, we lack knowledge of peptic ulcer history as well as preoperative PPI use and smoking habits at time of MU diagnosis, three factors demonstrated to be of importance in gastric bypass patients [38]. Finally, the low number of endoscopically verified MU is a limitation; however, in all patients, a formal diagnosis of MU was set in medical charts.

In conclusion, the incidence rate of marginal ulcers after duodenal switch and gastric bypass was low, around 1%, and did not differ between the two procedures. We believe that this is reassuring for future bariatric surgery candidates as well as bariatric surgeons.

## Electronic Supplementary Material

ESM 1 (DOCX 12 kb)
