# Supplementary material for: Low Risk for Marginal Ulcers in Duodenal Switch and Gastric Bypass in a Well-Defined Cohort of 472 Patients
Source: Obes Surg. 2020 Jul 8;30(11):4422–7. doi: 10.1007/s11695-020-04822-8 (PMC7524689; doi:10.1007/s11695-020-04822-8)
Supplement: Supplementary file 1 — (DOCX 12 kb) [file 11695_2020_4822_MOESM1_ESM.docx]

Supplementary table 1. Questionnaire for the survey of marginal ulcers after primary duodenal switch and gastric bypass, in patients with BMI >48.

1. Have you had a marginal ulcer after your bariatric surgery?

□ Yes

□ No.

2. If the answer is yes, how did you get the marginal ulcer diagnosis?

□ I underwent gastroscopy (examination of the stomach by a special instrument with camera)

□ My doctor set the diagnosis based on epigastric pain (without gastroscopy)

3. Have you been treated with any of the following drugs due to marginal ulcers after your bariatric surgery?

□ Omeprazole, Esomeprazole, Nexium, Lanzo, Lanzoperazole or Pantoprazole

□ Other preparation: ---------------------------------------------- -------------------------

4. Do you have ongoing treatment with any of the above-mentioned drugs?

□ Yes

□ No

5. If you answered yes to question 4, please tick one of the following boxes concerning the reason for the treatment.

□ Marginal or gastric ulcer

□ Heartburn or acid reflux

□ Other reasons: ---------------------------------------------- ---------------------------------------

6. Are you a current smoker?

□ Yes, I smoke more than one packet per day

□ Yes, I smoke less than one packet per day

□ No.
